# Which are the ‘Hilbert Problems’ of Biophysics?

**DOI:** 10.1017/qrd.2020.15

**Published:** 2021-01-05

**Authors:** Bengt Norden

**Affiliations:** 1 Chair of the Board of Editors, QRB Discovery; 2 Chair Professorship of Physical Chemistry, Department of Chemistry and Chemical Engineering, Chalmers University of Technology, SE-41296 Gothenburg, Sweden

Whilst considering commissioning articles for QRB Discovery as Editor-in-Chief, I would like to explain what I think could be interesting topics for new articles in addition to unexpected amazing discoveries.

Recently, I submitted a somewhat philosophical paper with the title ‘The Mole and Albert Einstein’ to which I had been invited by the Swedish Physics Society. To prepare myself, I had been reading Einstein’s famous papers and PhD Thesis of 1905 (in German to avoid interpreters’ pedagogical improvements!) and was struck by his quite narrow approach in his attempt to address and generalize so many different problems. I do not want to diminish the impression of his genius, but I suddenly saw Einstein as a somewhat bewildered young (25 years) man eager to make an impact. When in 2005 the Physics world celebrated the anniversary of Einstein’s miracle year, he was claimed to be a Chemist by Nature columnist Philip Ball because of his work and PhD Thesis on molecular diffusion inspired by Brownian motion and his goal to determine Avogadro’s number – but then why not call Einstein a Biophysicist?

Following van’t Hoff, the first Nobel Laureate in Chemistry (1901), Einstein starts with the osmotic pressure and van’t Hoff’s formula pV* = *nRT*, which looks like the ideal gas law, and which he assumes to be a thermodynamic macroscopic equation of state integrating corresponding microscopic equations in which the colliding solvent molecules just balance the osmotic pressure. His analysis (including some strange detours) leads to the famous statistical error function for diffusion. His explanation of the Brownian motion also results in the final acceptance by the science society of the atom and molecule concepts in the shape we know them today. The jumpy random motions that Robert Brown observed pollen grains to perform when suspended in water, Einstein explains, reflect the thermal motions of surrounding water molecules, an unequal number obviously colliding from opposite sides of the grain, telling something about the size of the numbers. The molecular dynamics and molecular-kinetic theory of heat are at the heart of Einstein’s thinking in 1905 and many years later his endeavor to develop a universal theory for the fundamental forces can be seen as just an expansion of this early work on liquids and intermolecular forces, based on Newtonian kinetic theory of matter.

Where am I heading with this? Well, I might ask: would a modern approach by molecular dynamics computation exactly agree with Einstein’s description of Brownian dynamics? Or might there be some deviations that could suggest additional, yet undiscovered mechanisms, such as wave front-like attacks by many coherently coupled solvent molecules by which the momentum transfer between the small molecules and big pollen grains could occur? The biophysical impact of such a mechanism could be earth-shattering, changing the rules for thermal activations of various processes inside the cell, adding large fluctuation forces. Unfortunately (or fortunately?), my checks in recent literature all indicate excellent agreement between modern molecular dynamics simulations and Brownian motion according to the classical Langevin equation as characterized in terms of autocorrelation memory and fluctuating force functions. Nor do any reported disagreement with experiment suggest the existence of any additional effects, neither under thermodynamic equilibrium conditions nor at investigated nonequilibrium conditions (electrophoresis and sedimentation). From this, I conclude that I must probably dismiss *fundamentals of molecular diffusion* as a problem area worth prioritized attention. Or maybe not since MD today is still limited to rather short times?

With this example, I would like to open a discussion asking: can we identify problem areas or paradigmatic models in biophysics and molecular biology that would deserve special attention because they are particularly important and not fully understood? Of course, we may not predict discovery, but as Pasteur says we could help chance for making one by having a prepared mind.

With inspiration from ‘Hilbert’s 23 Problems’, I would encourage all our readers, Editors, and Editorial Board Members to come up with suggestions for a list of such problems in contemporary biophysics-related sciences, whether theoretical, experimental, or intrinsic to the dissipative systems of living organisms. But like in my example above, before a certain problem be put forward, I would expect that effort has been made to search for evidence in the literature of any violations or conflicting data that can motivate further digging.

I shall list examples here, starting with fundamentals of mechanisms in biology and then theoretical challenges and potential future method developments. I welcome your adding more points to the list or complementing/correcting the current ones. We might also eventually do a scoring of the numbers in order of falling priority and to repeat this exercise when we have a more comprehensive list. Several problems are tough – if solved each likely to yield a Nobel Prize!

We would especially welcome authors of exciting reports related to any of the problem fields to submit contributions to *QRB Discovery*!

1. **Origin-of-life biophysics**. How can molecular model systems and simulations give insight into possible events, interactions, and structures that led up to what we may call ‘life’? All the way from the simplest elementary molecular reactions to complex molecular systems with function. How were lipids and membranes first made during the chemical origin of life? How does chirality determine interactions of molecules of life? Can chiral bias by parity violation of electroweak interactions be amplified by e.g. helical crystallization errors or spin-orbit coupling?
2. **Liquid–liquid phase transitions in cells and membrane-free organelles**. Roles in regulating life processes? Enthalpy-entropy compensation – frequently observed in biomolecular systems – potentially an effect of microscopic phase transitions (0 = Δ*H* − *T*Δ*S*)?
3. **Designer organisms and engineered enzymes.** Organisms with a minimal genome might become a tool for systematic studies of fundamental biological mechanisms and functions, and for producing bionic devices. Likewise designer proteins, in particular membrane protein design, may be exploited for making new bionic devices, including design of enzymes and design of whole cells. In the outskirts of biophysics, artificial DNA constructs (PNA) and DNA origami provide new challenges with applications outside biology. Using DNA-editing devices (CRISPR technology), one may envisage ‘DNA computation’.
4. **Memory enigma**. What is the molecular basis of learning and memory? Is long-term memory at all molecular? Maybe long-term memory is using the same reading machinery as inherited ‘basic instincts memory’ does? Perhaps in shape of proteins over-expressed and ‘preserved’ as amyloid aggregates (with >100 years lifetime)? On the other hand, short-term memory may be completely different and e.g. related to nonmolecular, macroscopic dendrite imprints recognizable when revisited upon new dendrite growth? Holistic/neural network aspects? Engram neuron cells?
5. **Enigma of protein folding**. How is misfolding and irreversible energy traps avoided? Steered folding: can one learn from experimental (designed model molecules) and theoretical models how enthalpic and entropic effects funnel the folding? The entropy seems notoriously hard to determine, a problem in molecular computations – could one develop some generic solution besides brutal force based on long and repeated simulations? Folding of partially synthesized protein emerging from ribosome may kinetically violate Anfinsen total length folded protein (rolling snowball encapsulation) by some Darwinian short-cuts?
6. **Machine learning and AI**. How help understand protein folding? Can AI help us develop molecular modeling programs without losing track of mechanisms and our physical understanding?
7. **Hydrophobicity and water**. In biology, hydrophobic effects lie behind the self-assembly and structuring of nucleic acids, folding of proteins, and aggregation of lipids into cell membranes. Despite the great importance of hydrophobic interactions, theoretical approaches do not seem able of providing any unifying picture of energetics and activation barriers to explain dynamics and catalytic effects of hydrophobic agents on structural changes and transport processes (such as motion within or crossing of a lipid membrane). Are there hydrophobic mechanisms that are not yet understood in terms of current theory or are they problematic just because of lack of sufficient free-energy accuracy (ΔG noise)? See also liquid–liquid phase transitions.
8. **Incomplete description of water**. How can we explain: Electrolyte ion-pair-specific bubble–bubble fusion interactions. The Hofmeister effect. Dissolved gas and microbubbles effects. ‘Nano-bubbles’, do they exist?
9. **Nonequilibrium biophysics**. Are current near-equilibrium models and short-time simulation tools insufficient for handling real nonequilibrium life processes? The theorems of Jarzynski and Crooks on thermodynamics and fluctuating forces suggest potentially new approaches to understand mechanisms of biological motors. Can collective motions or funneled transfer of momentum, such as soliton-like waves along a DNA coil, lead to large-amplitude fluctuations and energic ‘hot spots’ with roles in context of activation? Could vacuum shear slip overcome viscous damping? Can we develop generic models (shortcuts) to help us estimate entropy effects in molecular dynamics computations?
10. **Molecular motors**. Molecular mechanisms of muscle contraction, cell/cargo transport and locomotion, flagellar motion, DNA replication and repair. Virus DNA capsid packaging motors. Energy landscapes, use of ATP hydrolysis, and powering of conformational change/locomotion; F_o_F_1_-ATPsynthase chemical-mechanic energy conversion. Polymerization motors: tubulin/microtubule, GTP, directionality. One overarching principle? Catalytic steps? Ratchet models, thermal noise fluctuations, and reversibility?
11. **Ion channels and neuroscience.** Selectivity filters, ligand- and voltage-gated mechanisms. Proton channels. Receptor-mediated endocytosis mechanism. Evolution, selectivity, structure, and function – toxins and disease.
12. **Mechanism of olfaction.** What is its biophysical and molecular basis – from receptor to nerve signal? How may signal strength variations between different receptors and the corresponding spectrum of substance–signal strength combinatorially code for a highly resolved ‘smell fingerprint’? May this function principle be used technologically in chemical analysis or in molecular computers? What is the evolutionary relationship between olfactory receptors and internal neural signal receptors?
13. **Amyloids**. How do amyloid fibers form and what triggers their formation? How do their aggregation and degradation occur in cells? What is cause and what is consequence in the macroscopic aggregate formation, and what is its mechanistic relation to disease? Can aggregation be steered, safely inhibited or else controlled in a useful way? How are macroscopic hydrophobic and dielectric effects involved? Relation to prions? When do amyloids turn out to have infectious properties (‘turning bad’)? Intercellular transport by endocytosis?
14. **Cellular interactions and dynamics**.
  - **Coherent motion** of molecular reactions and flow. Membrane flow and cell migration. Interphase chromatin dynamics. Mechano-sensing.
  - **Crowding**. What are chemical (molecular interactions), steric (mechanical), and statistical (osmotic) effects?
  - **Communication between cells**. ‘Glycolipid crypticity’. Transfer of signal molecules and small RNAs, etc.
  - **Cell-membrane penetration mechanisms.** Cell-penetrating peptides. Molecular partners annihilating polarity and lowering Born barrier. Endocytosis. Organelle-specific delivery to cancer cells for therapy.
15. **Quantum biology**. While in light-harvesting organisms, excitonic states are trivially quantum entangled pairs of excited states, it has been claimed that quantum entanglement may also lie behind amplifications of extremely tiny radical interactions and explain e.g. birds’ ability to navigate in the nighttime in Earth’s magnetic field. Does such quantum biology really exist i.e. are there any specific processes explainable only as quantum phenomena?
16. **Electric and magnetic biology**. What is the origin of macroscopic electric and magnetic effects exhibited by bacteria? ‘Electrets’ are immobilized electric charges at surfaces but neither creation nor dielectric stabilization of an electret is well understood? ‘Magnetosomes’ as artificial magnetizable cell models?
17. **High hydrostatic pressure biophysics**. Organism adaptions and habitat changes in deep sea, lipid desaturase activation, metabolic adjustment. What can be learnt from high-pressure experiments about local structures and phase transitions in cells?
18. **Methodology and translational science-driven development**
**Structure/morphology at multiple scales**. Advances in technology e.g. visualization/imaging techniques, open new biophysical avenues.
  - Atomic scale (electron, X-ray, including X-ray free-electron laser)
  - Supramolecular scale (cryo-EM)
  - Subcellular/cellular (cryo-ET and other high-resolution techniques)
**Dynamics at multiple scales**. Not sufficient with spatial/structural/morphological view only, but their time evolution is needed too. Both experimental and computational techniques/models/simulations that examine time-dependent changes in structures (at multiple scales) are important. Dynamics is the bridge between structure and function.
**System biology**. Challenge to bridge experiments and theory. Theories/models that can be experimentally tested or take advantage of new data to build/improve new/existing models. ‘Systems-level’ approaches that provide a better understanding of systems dynamics in its physiological environment. Quantitative systems pharmacology typical example.
**4D genome.** Ability today to do structure-based studies at the level of chromosomes or the entire chromatin. With advances in Hi-C technology, we now have 3D connectivity information on gene loci, which may be used for generating 3D structures, and even their equilibrium fluctuations (modes of motions using linear theory). So, do not think of DNA as a string of letters with a code, but as a 4D entity.
**Data-driven studies, machine learning, artificial intelligence**. Such studies are extremely powerful, and papers on this area should be given serious consideration, provided that the utility of the method is illustrated/documented by concrete biological examples. Approaches that combine machine learning/AI methods and physical sciences-based methods, or those that allow for physical interpretations of AI predictions, should be encouraged.
**Cryoelectron Microscopy.** Future developments from specimen preparation, electron energy, electron optics, detector, and image processing? Discovery of discrepancies revealing perturbation effects in the crystal state? Development of liquid cell system to image proteins/viruses in solution. Determining protein structure inside cells (including use of correlated light and electron microscopies).
**Free-electron laser x-ray diffraction**. Future developments? Is single protein molecule diffraction really possible?
**Single molecule studies**. Advantage to ensemble averaging: a single molecule is always perfectly macroscopically oriented! Rate studies: a molecular conformational transition back and forth can provide both statistical (thermodynamic) and dynamic (kinetic rate constants) data in one experiment. Today’s tools include various forms of pulling-force spectroscopy and fluorescence correlation microscopy. Anticipated new tools in the future?
**Microfluidics.** and related methods connecting different spatial and temporal resolution scales for example molecular models, biomimics, and tissue samples. Complement transport with chemical reaction stations using covalent surface immobilization into microscopic assembly lines.
**Density functional theory**. According to a theorem by Kohn and Hohenberg, an electron density functional always exists that can electrostatically uniquely define a molecular ground state without need for consideration of Schrödinger wavefunctions. However, despite this existence proof, nobody has yet been able to produce such a density functional, and so-called DFT methods do not rest on true Density Functionals.

